# Concerns about the interpretation of treatment effects

**DOI:** 10.1128/aac.00695-24

**Published:** 2024-07-16

**Authors:** Shougen Sumiyoshi

**Affiliations:** 1 Division of Infection Control and Prevention, Osaka University Hospital, Osaka, Japan; University of Fribourg, Fribourg, Switzerland

**Keywords:** ocular syphilis, ceftriaxone, penicillin

## LETTER

I appreciate the efforts of Gu et al. in conducting an interesting study (1), wherein they deduced that ceftriaxone was as effective as penicillin for treating ocular syphilis and indicated that ceftriaxone may be a viable primary treatment. Our concern, however, is the interpretation of the results of this study.

They used the propensity score weighting and revealed no significant intergroup difference in treatment efficacy, as indicated by non-significant *P*-values. However, compared to the ceftriaxone group, the penicillin group appears to have a numerically higher proportion of “effective” results. A small sample size necessitates caution, and the standardized mean difference, rather than *P*-values, may be more appropriate to compare intergroup differences (2, 3). In addition, this study should be based on a non-inferiority design, but the non-inferiority margin is not predefined. This complicates the interpretation of the results.

Thus, despite the various advantages of ceftriaxone, further examination is required before the results of this study can be applied in clinical practice.
